# Definition of healthcare‐associated influenza: A review and results from an international survey

**DOI:** 10.1111/irv.12460

**Published:** 2017-07-18

**Authors:** Elodie Munier‐Marion, Thomas Bénet, Philippe Vanhems

**Affiliations:** ^1^ Infection Control and Epidemiology Unit Hospices Civils de Lyon Edouard Herriot Hospital Lyon France; ^2^ Emerging Pathogens Laboratory – Fondation Mérieux INSERM U1111, CNRS, UMR5308, ENS de Lyon, UCBL1 Centre International de Recherche en Infectiologie Lyon France; ^3^ INSERM, F‐CRIN, I‐REIVAC Lyon Collaborative Center Lyon France

**Keywords:** definition, healthcare‐associated, influenza

## Abstract

**Aim:**

To describe definitions of healthcare‐associated influenza (HAI) in recent literature and in hospitals participating in a survey of Society for Healthcare Epidemiology of America (SHEA) Research Network (SRN) members.

**Method:**

A review with PubMed search was undertaken to retrieve articles published between 2008 and 2016, focusing on the subject headings “influenza, human” and “cross infection.” Definitions of clinical influenza‐like illness (ILI) and HAI were identified. An invitation to participate in the survey was sent to 218 SRN members via email.

**Results:**

Of 75 articles on HAI included in the review, 30 presented a standardized definition of clinical ILI based on fever (100%), cough (80%), and sore throat (70%). Forty studies (53%) contained a standardized HAI definition, grounded on threshold delay from admission in 29 of them, this delay ranging from 48 to 196 hour (median: 72 hour). Fifty‐five SRN members responded to the survey, with a standardized definition of HAI adopted by 76% of them. This definition was based on clinical features for 24%, virological features for 31%, and both for 45%. Fever (mean threshold: 38.0°C) was part of the definition for 82%. The features required most frequently in the clinical definition were cough (46%) and sore throat (26%). Median threshold delay between admission and symptoms onset adopted for HAI definition was 48 hour (range: 24‐96 hour).

**Conclusion:**

This work underlined the heterogeneity of HAI definitions in different countries. A standardized definition would be helpful to evaluate HAI spread, outcomes in patients and healthcare systems, and the impact of prevention measures, including vaccination.

## 1 INTRODUCTION

Healthcare‐associated influenza (HAI) is associated with significant morbidity, mortality, and costs attributed to increased length of stay, but is likely to be under‐recognized.^1^ Early detection leads to rapid implementation of respiratory isolation precautions and prevents nosocomial transmission between patients and healthcare personnel (HCP). Timely recognition could obviate extra testing and treatment. The need for a standardized definition of HAI has been underlined previously.^2^ A synthesis using the Outbreak Reports and Intervention Studies of Nosocomial Infection (ORION) statement highlighted the dearth of standardized information collected during HAI outbreak investigations,^3^ which limits comparability between studies. Although standardized definitions of clinical influenza‐like illness (ILI) were implemented in some observational and interventional studies, they differed from one another in terms of body temperature threshold, symptoms, and time interval between hospitalization and symptoms onset.^3^

The World Health Organization, the Centers for Disease Control and Prevention (CDC), and the European Center for Disease Prevention and Control have proposed different definitions of community ILI.^4^, ^5^, ^6^ To control the spread of HAI, definition of clinical cases should afford high sensitivity to avoid cases being missed.^3^ To the best of our knowledge, no review has systematically reported clinical and virological criteria defining HAI. Reported HAI and contemporary definitions used by HCP might help to improve the reliability and homogeneity of HAI definition.

The purpose of this study was to report HAI definition: (i) in recent literature and (ii) in hospitals participating in the SHEA (*Society for Healthcare Epidemiology of America*) Research Network (SRN). The results might help to facilitate guideline updating on HAI and collaborative surveys and studies.

## 2 METHODS

A literature review with PubMed search was undertaken with the medical subject headings (MeSH) “influenza, human” and “cross infection” to find English‐ and French‐language articles published between January 1, 2008, and June 1, 2016. A previous synthesis of the literature done with similar MeSH terms by our team for articles published before 2008 had other objectives; these studies were not included in this review.^3^ Titles and/or abstracts were analyzed to trace publications on influenza or ILI infections in hospital settings. Articles with healthcare‐associated influenza or ILI cases as outcome were selected. Definitions of clinical ILI and HAI were described in selected articles. Country, study period, study design, population involved (patient and/or HCP) were also gathered.

An invitation to participate in the online survey was sent to all SRN members via e‐mail on July 9, 2015, followed by two e‐mail reminders. The survey was posted publicly on the SRN Web site (http://www.shea-online.org). The SRN is a consortium of more than 200 hospitals worldwide, with collaborative multicenter research projects in healthcare epidemiology.^7^ The questionnaire asked about the demographic characteristics of respondent hospitals, definitions of clinical ILI and HAI, recommended tests, detection of clusters and notification (see Data S1). Additional questions were asked concerning a particular definition to clinically suspect influenza in patient with clinical ILI. Statistics were reported as means (range), medians, and percentages to describe the study results.

## 3 RESULTS

### 3.1 Review of the literature

Seventy‐five (26% of the 292 articles of the research) articles on HAI were included in the review. Table S1 describes the place, period, study design, population, ILI definition, HAI definition, and references. In terms of design, 28 (37%) were prospective studies, 23 (31%) were retrospective, 21 (28%) were outbreak reports, two (3%) were control trials, and one (1%) was quasi‐experimental. Patients were selectively included in 43 (57%), HCP in eight (11%), and both populations in 24 (32%) studies.

**Figure 1:**
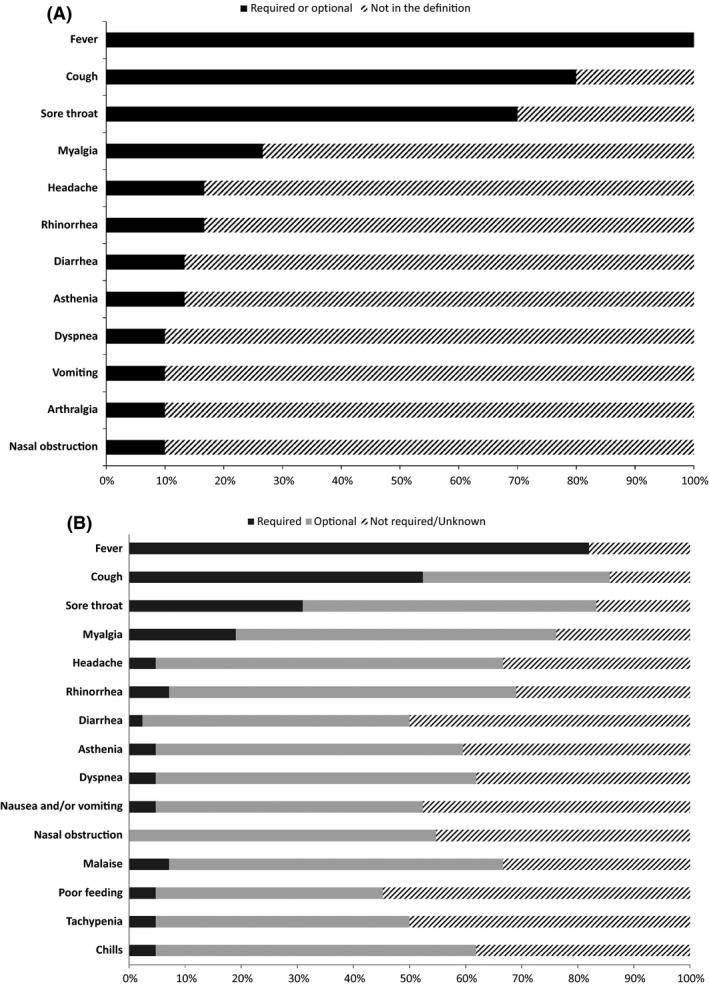
(A) Clinical features present in at least three ILI patient definitions: a literature review (N=30). (B) Clinical features present in ILI patient definition: survey of Society for Healthcare Epidemiology of America (SHEA) Research Network members (N=42)

**Figure 2:**
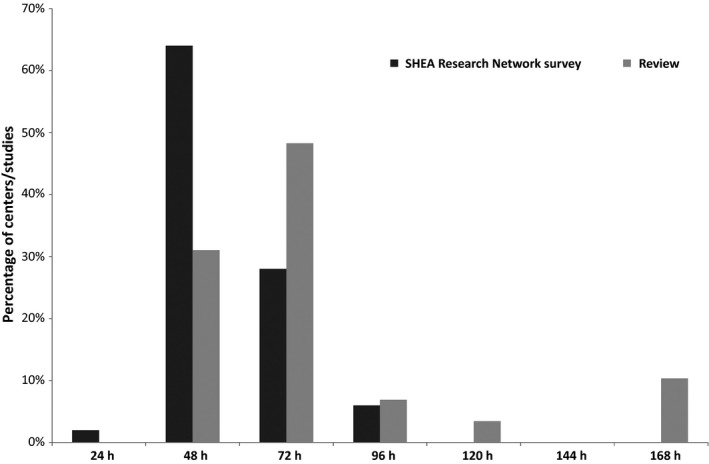
Threshold delay between admission in hospital/unit and symptoms onset in HAI definition: a literature review and survey of Society for Healthcare Epidemiology of America (SHEA) Research Network (SRN) members

A standardized definition of clinical ILI was given in 30 studies (40%). This definition required at least the presence of fever in all them (Figure 1A). A fever threshold was defined in 19 studies (mean 37.9°C; range 37.5‐38.0°C). Cough and sore throat were present in the definition in 24 and 21 studies, respectively (80% and 70% of those with definition). The association of different signs/symptoms (fever/cough/sore throat) was noted in 70% of definitions. The definition was less precise in 16 studies (21%): It comprised the following terms: “influenza‐like illness,” “respiratory infection,” “acute respiratory illness,” “signs and symptoms of influenza.” Symptoms presented by included cases were reported but without case definition in 12 outbreak investigations. No definition of clinical ILI was seen in 17 studies (23%): Definition in these studies was based on positive virological results. Two studies had different definitions for patients and HCP. In one study, the definition was adapted for the elderly, for patients with mental disorders, or in the context of outbreaks. Forty studies (53%) implemented a standardized definition of the healthcare‐associated (HA) nature of these infections. This definition was based on the minimum threshold delay between hospital admission and symptoms onset in 29 studies (73%) with variations from 48 to 196 hour (mean: 78 hour; median: 72 hour; Figure 2). The definition was linked with symptoms onset after admission but without a determined threshold in five (13%) studies and contact with a contagious individual in six studies (15%). Three studies were conducted in neonatal units where newborns were hospitalized since their birth. Overall, no criterion for nosocomial acquisition was given in 32 studies (43%).

### 3.2 SRN survey

Fifty‐five of 218 SRN members solicited responded to the survey (response rate: 25%). Respondents in the survey were from North America (82%), Asia (7%), Europe (4%), South America (2%), and Australia (2%); the origin of two respondents was unknown. Seventy‐five percent of respondents were hospital epidemiologists and 19% infection preventionists; 78% of healthcare facilities were affiliated with academic institutions.

Overall, 76% of respondents worked with a standardized definition of HAI (N=42). This definition was based on clinical features only for 24%, on virological elements only for 31%, and on both categories for 45% (Table 1). Fever was part of the definition for 82% of those who had a standardized definition. The mean minimal threshold defining fever in patients and HCP free of antipyretics was 38.0°C (range: 37.0‐39.0°C). The clinical features mostly required in the definition of ILI patients were cough (52%), sore throat (31%), and myalgia (19%, Figure 1B). Thirty‐six percent of respondents used a particular definition to clinically suspect influenza in patient with clinical ILI. Among them, 94% took fever into account in their “clinical influenza” definition. Cough, sore throat, and myalgia were required symptoms in 75%, 45%, and 35% of respondents, respectively. Virological testing consisted of throat swabs for 2% of respondents, nasal or nasopharyngeal swabs for 69%, and swabs from both sites for 29%. The most frequently performed tests were molecular identification (RT–PCR, 77%) and antigen detection by rapid diagnostic test (19%). Virological samples were sent to a national reference center in 38% of healthcare settings. The mean minimum threshold delay between admission in the unit and symptoms onset defining HAI was 57.1 hour (median: 48.0 hour; Figure 2). Another criterion often identifying HAI was contact with symptomatic persons. Nineteen percent of respondents (N=8) cited an adapted definition of HA‐ILI and/or HAI for immunocompromised patients (N=8), patients with hematological malignancies (N=3), cancer (N=3), HIV/AIDS (N=3), and the elderly (N=1). Seven percent had different definitions of HA‐ILI and/or HAI for HCP and patients. Forty‐three percent adopted a standardized definition for cluster identification of HAI. The median number of cases defining a cluster was 3. HCP were counted in the cluster by 78% of respondents. Similarity of strains was needed to confirm a cluster for 43% of respondents. Sixty‐two percents reported HAI cases to other institutions, to their institution for 47% of them, to local public health authorities for 88%, and to national public health authorities for 6%.

**Table 1:** Healthcare‐associated influenza‐like illness and influenza definition used by Society for Healthcare Epidemiology of America (SHEA) Research Network members (N=55)

	% (N/N respondent)
Definition of HA influenza	76% (42/55)
Definition based on	
Clinical features	24% (10/42)
Virological features	31% (13/42)
Both	45% (19/42)
Clinical features of ILI	
Fever part of ILI definition	82% (32/39)
Mean threshold for patients without antipyretics (range)	38.0°C (37.0‐39.0°C)
Clinical features of influenza	
Appropriate clinical features for early detection of influenza	36% (20/55)
Fever part of influenza definition	94% (17/18)
Mean threshold for patients without antipyretics (range)	38.1°C (37.8‐39.0°C)
Virological features	
Swabs realized	
Nasal/nasopharyngeal swabs	69% (35/51)
Throat swabs	2% (1/51)
Both	29% (15/51)
Tests performed	
RT–PCR	77% (40/52)
Rapid diagnostic test	19% (10/52)
Virological samples sent to national reference center	38% (20/52)
HA definition	
Mean threshold delay between admission in the unit and symptoms onset for HA influenza definition (range) in h	57.1 (24.0‐96.0)
Threshold delay used	
>12 h	0% (0/50)
>24 h	2% (1/50)
>48 h	64% (32/50)
>72 h	34% (17/50)

The denominator for percentages is based on respondents to the question.

ILI, influenza‐like illness; HA, healthcare‐associated

## 4 DISCUSSION

This review and prospective survey underlined differences in HAI definitions between centers in different countries and continents. Not surprisingly, fever was frequent in HAI definitions, but the temperature threshold was heterogeneous. Cough and sore throat were the other most common symptoms present in HAI definitions. Delay between admission and symptoms onset should be one of the major criteria defining HA cases. However, this threshold ranged from 48 hour to 7 days in the review and 24‐96 hour in the survey.

Different local definitions might be justified by variability of infection control practices or populations, but the absence of a standardized delay could generate some difficulties which might lead to under‐ or overestimate the HAI burden, misclassify community or healthcare‐associated influenza, and, finally, impact hospital‐attributable risk and control of disease spread. This delay is related to the incubation period; however, pooled median incubation periods of influenza A and B were shorter (1.4 and 0.6 days, respectively), with 95% of patients infected by influenza A and B, respectively, developing symptoms in 2.8 and 1.1 days after infection.^8^ In clinical practice, the threshold to define an influenza case as HA would be different from incubation period of the disease and could vary according to objectives. By increasing this delay, specificity of the definition would be improved but sensitivity would be lower. For example, the definition for surveillance might differ from the definition for outbreak investigations. Moreover, some healthcare settings used different definitions for some categories of patients (ie immunocompromised, the elderly), which limited comparison. This heterogeneity is, however, justified because of different clinical ILI presentations. Several national surveillance networks exist, but the HAI definitions given are different. For example, HAI definition is based on laboratory surveillance and on a delay of 48 hour in Australia^9^ compared to 96 hour in Canada.^10^ In our hospital in France, surveillance is founded on ILI identification (clinical definition) with secondary virological confirmation, we include all ILI cases, without taking time from admission into account.^11^ Rapid diagnostic tests are helpful for diagnostic confirmation, rapid implementation of control measures, for proposing antiviral treatment, and avoiding antibiotics prescription. Absence of virological confirmation could lead to the implementation of inadequate measures in case of outbreaks due to other respiratory viruses. Sensitivity of these tests is, however, not optimal,^12^ and they do not resolve the issue of clinical ILI identification. In addition, practices vary widely between hospital settings and contexts, which might result in low detection rates. The issue of asymptomatic infected persons has to be raised. Indeed, the asymptomatic fraction might range from 4% to 28% of patients.^13^ The characteristics of hospitalized patients, such as concomitant medications or underlying illnesses, could partially explain the lack of correlation between symptoms and influenza infections. A high index of suspicion and awareness of less expected presentations is necessary during influenza seasons to facilitate the rapid identification of infected patients. Indeed, in a US study, only 51% of patients who received a laboratory‐confirmed diagnosis of influenza met CDC surveillance criteria.^14^ In a prospective study, both sensitivity and the positive predictive value of fever and cough in the diagnosis of influenza virus infection were low in hospitalized patients (35% and 23%, respectively).^15^

One of a main added value of this study is its international perspective, including countries from both hemispheres, which increased external validity. These results underscored the need for rigorous methodology in international studies including clinical trials and bundle implementation for HAI control. In addition, if standardization is too challenging, the results highlight the need for appropriate statistical analyses. A study limitation was the modest response rate, which was, however, similar to that in previous SHEA studies. There was also a response bias as only SRN members were asked to participate. The determinants of differences in HAI definitions should be explored more in depth in the future. In addition, study power was limited to stratify analysis by country and region. Concerning the review, the main limitation is the heterogeneity of study design which precludes quality evaluation with standardized scale as PRISMA.^16^ However, our primary objective was to include every study (surveillance, outbreak investigation…) to focus on ILI or influenza case definition and to be as exhaustive as possible. We did not aim to evaluate the impact of case definition on study result. In addition, our review was done on a unique data base (PubMed), and we cannot exclude that some studies are missing. However, MeSH terms guarantee a better efficiency of the search, as they are highly sensitive for a literature review.^17^

In conclusion, we found evidence that HAI definitions varied widely. A standardized HAI definition would be helpful in evaluating HAI spread, cluster investigation, attack rate comparisons, evaluating the consequences to patients and healthcare systems, particularly legal and assurance outcomes, intervention and prevention impacts, including vaccine effectiveness. Future studies might generate algorithms classifying HAI as proven, probable or possible, while taking clinical presentation, virological findings, and delay between hospital admission and symptoms onset into account.

## CONFLICT OF INTEREST

There are no conflicts of interest.
