# Supplementary material for: Definition of healthcare‐associated influenza: A review and results from an international survey
Source: Influenza Other Respir Viruses. 2017 Jul 18;11(5):367–71. doi: 10.1111/irv.12460 (PMC5596525; doi:10.1111/irv.12460)
Supplement: Supplementary file 1 [file IRV-11-367-s001.doc]

#### Supplementary table. Description of studies included in review, N=75 studies

| **First author** | **Country** | **Study period** | **Study design** | **Population** | **ILI definition** | **HAI definition** |
| --- | --- | --- | --- | --- | --- | --- |
| Altmann^1^ | Germany | 2009-2011 | prospective | patients | no | symptoms onset >48 h after admission |
| Amodio^2^ | Italy | 2005-2012 | retrospective | patients | no | symptoms onset >48 h after admission |
| Apisarnthanarak^3^ | Thailand | 2004-2006 | prospective | patients + HCP | no | contact with a contagious case |
| Apisarnthanarak^4^ | Thailand | 2005-2009 | quasi experimental study | patients + HCP | no | contact with a contagious case |
| Bearden^5^ | USA | 2009 | outbreak report | patients + HCP | no |  |
| Bénet^6^ | France | 2004-2007 | prospective | patients | yes | symptoms onset >72 h after admission |
| Bhadelia^7^ | USA | 2009-2010 | retrospective | HCP | yes |  |
| Buchbinder^8^ | France | 2010 | outbreak report | patients | no |  |
| Chan^9^ | Hong Kong | 2011 | outbreak report | patients + HCP | no |  |
| Chen^10^ | USA | 2009 | outbreak report | patients + HCP | no |  |
| Cheng^11^ | Hong Kong | 2009 | prospective | patients + HCP | yes | symptoms onset >48 h after admission |
| Cheng^12^ | China | 2015 | prospective | HCP | no |  |
| Chironna^13^ | Italy | 2009 | outbreak report | patients | yes |  |
| Chu^14^ | Taiwan | 2009-2010 | prospective | HCP | yes |  |
| Cordero^15^ | Spain | 2009-2010 | prospective | patients | no | symptoms onset >7 days after admission |
| de-Paris^16^ | Brazil | 2007-2010 | retrospective | patients | no | symptoms onset after admission |
| Du^17^ | China | 2009-2010 | prospective | patients + HCP | no | symptoms onset >48 h after admission |
| Eibach^18^ | France | 2012 | outbreak report | patients + HCP | yes | symptoms onset >72 h after admission |
| Enstone^19^ | UK | 2009-2010 | retrospective | patients | no | symptoms onset >72 h after admission |
| Fanella^20^ | Canada | 2009 | retrospective | patients | no | symptoms onset >7 days after admission |
| Fang^21^ | China | 2015 | outbreak report | patients | no |  |
| Farooqui^22^ | China | 2015 | outbreak report | patients + HCP | no |  |
| Gaillat^23^ | France | 2005 | outbreak report | patients + HCP | yes |  |
| Gaspard^24^ | France | 2004-2012 | prospective | patients | yes |  |
| Gooskens^25^ | Netherlands | 2008 | outbreak report | patients + HCP | no |  |
| Greenbaum^26^ | USA | 2011 | prospective | HCP | yes | contact with a contagious case |
| Haber^27^ | France | 2005-2006 | prospective | patients | no | symptoms onset >48 h after admission |

#### Table 1, continued.

| **First author** | **Country** | **Study period** | **Study design** | **Population** | **ILI definition** | **HAI definition** |
| --- | --- | --- | --- | --- | --- | --- |
| Higa^28^ | Japan | 2007-2010 | retrospective | patients + HCP | no | contact with a contagious case |
| Hui^29^ | Hong Kong | 2006-2007 | prospective | patients | yes |  |
| Huzly^30^ | Germany | 2013-2014 | retrospective | patients | yes | symptoms onset >72 h after admission |
| Jaeger^31^ | USA | 2009 | prospective | HCP | yes | contact with a contagious case |
| Jhung^32^ | USA | 2010-2011 | retrospective | patients | no | symptoms onset >72 h after admission |
| Jonges^33^ | Netherlands | 2009 | retrospective | patients + HCP | no | symptoms onset >48 h after admission |
| Khandaker^34^ | Australia | 2009 | prospective | patients | no | symptoms onset >72 h after admission |
| Lalayanni^35^ | Greece | 2009 | outbreak report | patients | no |  |
| Launay^36^ | Canada | 2009 | retrospective | patients | yes | symptoms onset >72 h after admission |
| Leckerman^37^ | USA | 2000-2004 | retrospective | patients | no | symptoms onset >72 h after admission |
| Levin-Rector^38^ | USA | 2013-2014 | prospective | patients | no |  |
| Liu^39^ | China | 2009-2010 | prospective | patients + HCP | yes |  |
| Liu^40^ | USA | 2009 | retrospective | patients | no |  |
| Lobo^41^ | Brazil | 2009 | prospective | HCP | yes |  |
| Loeb^42^ | Canada | 2008-2009 | clinical trial | HCP | yes |  |
| Macesic^43^ | Australia | 2010-2011 | prospective | patients | no | symptoms onset >48 h after admission |
| Mahmud^44^ | Canada | 2003-2011 | retrospective | patients + HCP | yes |  |
| Marzano^45^ | Italy | 2011 | outbreak report | patients | no | symptoms onset >48 h after admission |
| Miller^46^ | UK | 2009 | outbreak report | patients + HCP | no |  |
| Milupi^47^ | UK | 2010 | outbreak report | patients | no | neonatal unit |
| Mitchell^48^ | Canada | 2006-2011 | prospective | patients | yes | symptoms onset >96 h after admission |
| Moore^49^ | UK | 2009 | outbreak report | patients | yes |  |
| Nivin^50^ | USA | 2004-2006 | prospective | patients | no |  |
| Oguma^51^ | Japan | 2007 | retrospective | patients + HCP | yes | symptoms onset >72 h after admission |
| Pagani^52^ | Switzerland | 2012 | outbreak report | patients + HCP | yes | symptoms onset >72 h after admission |
| Perez-Padilla^53^ | Mexico | 2007-2009 | retrospective | patients | no |  |
| Pollara^54^ | Italy | 2011 | retrospective | patients | no | symptoms onset after admission |
| Rabagliati^55^ | Chile | 2009 | prospective | patients | no |  |

#### Table 1, continued.

| **First author** | **Country** | **Study period** | **Study design** | **Population** | **ILI definition** | **HAI definition** |
| --- | --- | --- | --- | --- | --- | --- |
| Régis^56^ | France | 2004-2009 | prospective | patients | yes | symptoms onset after admission |
| Reid^57^ | USA Canada | 2009-2010 | retrospective | patients | no | symptoms onset >7 days after admission |
| Riphagen-Dalhuisen^58^ | Netherlands | 2009-2011 | clinical trial | patients | no | symptoms onset >72 h after admission |
| Risa^59^ | USA | 2006 | outbreak report | patients + HCP | no |  |
| Rodriguez-Sanchez^60^ | Spain | 2009 | retrospective | patients | no |  |
| Rubin^61^ | USA | 2001-2004 | prospective | patients | no |  |
| Seale^62^ | Australia | 2005 | outbreak report | patients + HCP | yes |  |
| Spaeder^63^ | USA | 2002-2008 | retrospective | patients | no | symptoms onset >48 h after admission |
| Taylor^64^ | Canada | 2006-2012 | prospective | patients | yes | symptoms onset >96 h after admission |
| Tsagris^65^ | Greece | 2011 | outbreak report | patients + HCP | yes | neonatal unit |
| Valley-Omar^66^ | South Africa | 2011 | retrospective | patients | no | symptoms onset >72 h after admission |
| Vanhems^67^ | France | 2004-2007 | prospective | patients + HCP | yes | symptoms onset after admission |
| Vayalumkal^68^ | Canada | 2005 | prospective | patients | yes | symptoms onset >72 h after admission |
| Veenith^69^ | UK | 2010-2011 | retrospective | patients | no | symptoms onset >5 days after admission |
| Vij^70^ | USA | 2009-2010 | outbreak report | patients | no | neonatal unit |
| Voirin^71^ | France | 2012 | prospective | patients + HCP | yes | symptoms onset after admission |
| Weedon^72^ | USA | 2003-2010 | retrospective | patients | yes | symptoms onset >72 h after admission |
| Wilkinson^73^ | Canada | 2009-2010 | retrospective | patients | no |  |
| Wise^74^ | USA | 2009 | prospective | HCP | yes | contact with a contagious case |
| Wong^75^ | Hong Kong | 2008 | outbreak report | patients + HCP | no |  |

HCP: healthcare personnel; USA: United States of America; UK: United Kingdom

References:

1. Altmann M, Fiebig L, Buda S, von Kries R, Dehnert M, Haas W. Unchanged severity of influenza A(H1N1)pdm09 infection in children during first postpandemic season. Emerg Infect Dis 2012;18:1755-1762.

2. Amodio E, Restivo V, Firenze A, Mammina C, Tramuto F, Vitale F. Can influenza vaccination coverage among healthcare workers influence the risk of nosocomial influenza-like illness in hospitalized patients? J Hosp Infect 2014;86:182-187.

3. Apisarnthanarak A, Puthavathana P, Kitphati R, Auewarakul P, Mundy LM. Outbreaks of influenza A among nonvaccinated healthcare workers: implications for resource-limited settings. Infect Control Hosp Epidemiol 2008;29:777-780.

4. Apisarnthanarak A, Uyeki TM, Puthavathana P, Kitphati R, Mundy LM. Reduction of seasonal influenza transmission among healthcare workers in an intensive care unit: a 4-year intervention study in Thailand. Infect Control Hosp Epidemiol 2010;31:996-1003.

5. Bearden A, Friedrich TC, Goldberg TL, et al. An outbreak of the 2009 influenza a (H1N1) virus in a children’s hospital. Influenza Other Respir Viruses 2012;6:374-379.

6. Bénet T, Régis C, Voirin N, et al. Influenza vaccination of healthcare workers in acute-care hospitals: a case-control study of its effect on hospital-acquired influenza among patients. BMC Infect Dis 2012;12:30.

7. Bhadelia N, Sonti R, McCarthy JW, et al. Impact of the 2009 influenza A (H1N1) pandemic on healthcare workers at a tertiary care center in New York City. Infect Control Hosp Epidemiol 2013;34:825-831.

8. Buchbinder N, Dumesnil C, Pinquier D, et al. Pandemic A/H1N1/2009 influenza in a paediatric haematology and oncology unit: successful management of a sudden outbreak. J Hosp Infect 2011;79:155-160.

9. Chan MC, Lee N, Ngai KL, et al. A “pre-seasonal” hospital outbreak of influenza pneumonia caused by the drift variant A/Victoria/361/2011-like H3N2 viruses, Hong Kong, 2011. J Clin Virol 2013;56:219-225.

10. Chen LF, Dailey NJ, Rao AK, et al. Cluster of oseltamivir-resistant 2009 pandemic influenza A (H1N1) virus infections on a hospital ward among immunocompromised patients--North Carolina, 2009. J Infect Dis 2011;203:838-846.

11. Cheng VC, Lee WM, Sridhar S, Ho PL, Yuen KY. Prevention of nosocomial transmission of influenza A (H7N9) in Hong Kong. J Hosp Infect 2015;90:355-356.

12. Cheng VC, Tai JW, Wong LM, et al. Prevention of nosocomial transmission of swine-origin pandemic influenza virus A/H1N1 by infection control bundle. J Hosp Infect 2010;74:271-277.

13. Chironna M, Tafuri S, Santoro N, Prato R, Quarto M, Germinario CA. A nosocomial outbreak of 2009 pandemic influenza A(H1N1) in a paediatric oncology ward in Italy, October-November 2009. Euro Surveill 2010;15.

14. Chu TP, Li CC, Wang L, et al. A surveillance system to reduce transmission of pandemic H1N1 (2009) influenza in a 2600-bed medical center. PloS One 2012;7:e32731.

15. Cordero E, Pérez-Romero P, Moreno A, et al. Pandemic influenza A(H1N1) virus infection in solid organ transplant recipients: impact of viral and non-viral co-infection. Clin Microbiol Infect 2012;18:67-73.

16. de-Paris F, Beck C, Pires MR, dos Santos RP, Kuchenbecker R de S, Barth AL. Viral epidemiology of respiratory infections among children at a tertiary hospital in Southern Brazil. Rev Soc Bras Med Trop 2014;47:223-226.

17. Du M, Suo J, Jia N, Xing Y, Xie L, Liu Y. The cross-transmission of 2009 pandemic influenza A (H1N1) infections among healthcare workers and inpatients in a Chinese tertiary hospital. Infect Control Hosp Epidemiol 2012;33:295-298.

18. Eibach D, Casalegno JS, Bouscambert M, et al. Routes of transmission during a nosocomial influenza A(H3N2) outbreak among geriatric patients and healthcare workers. J Hosp Infect 2014;86:188-193.

19. Enstone JE, Myles PR, Openshaw PJ, et al. Nosocomial pandemic (H1N1) 2009, United Kingdom, 2009-2010. Emerg Infect Dis 2011;17:592-598.

20. Fanella ST, Pinto MA, Bridger NA, et al. Pandemic (H1N1) 2009 influenza in hospitalized children in Manitoba: nosocomial transmission and lessons learned from the first wave. Infect Control Hosp Epidemiol 2011;32:435-443.

21. Fang CF, Ma MJ, Zhan BD, et al. Nosocomial transmission of avian influenza A (H7N9) virus in China: epidemiological investigation. BMJ 2015;351:h5765.

22. Farooqui A, Liu W, Kelvin DJ, et al. Probable Hospital Cluster of H7N9 Influenza Infection. N Engl J Med 2016;374:596-598.

23. Gaillat J, Dennetière G, Raffin-Bru E, Valette M, Blanc MC. Summer influenza outbreak in a home for the elderly: application of preventive measures. J Hosp Infect 2008;70:272-277.

24. Gaspard P, Mosnier A, Gunther D, et al. Influenza outbreaks management in a French psychiatric hospital from 2004 to 2012. Gen Hosp Psychiatry 2014;36:46-52.

25. Gooskens J, Jonges M, Claas EC, Meijer A, van den Broek PJ, Kroes AM. Morbidity and mortality associated with nosocomial transmission of oseltamivir-resistant influenza A(H1N1) virus. JAMA. 2009;301:1042-1046.

26. Greenbaum AH, Wong K, Nguyen D, et al. Assessment for possible healthcare-associated transmission of a new variant influenza virus--Pennsylvania, August 2011. Infect Control Hosp Epidemiol 2013;34:1306-1309.

27. Haber N, Dekimeche S, Cantet C, Marquand D, Szekely C, Lebon P. [Lower respiratory tract infections with influenza and respiratory syncytial viruses in hospitalized elderly patients during the 2005-2006 winter season]. Presse Médicale Paris Fr 1983 2009;38:893-903.

28. Higa F, Tateyama M, Tomishima M, et al. Role of neuraminidase inhibitor chemoprophylaxis in controlling nosocomial influenza: an observational study. Influenza Other Respir Viruses 2012;6:299-303.

29. Hui DS, Woo J, Hui E, et al. Influenza-like illness in residential care homes: a study of the incidence, aetiological agents, natural history and health resource utilisation. Thorax 2008;63:690-697.

30. Huzly D, Kurz S, Ebner W, Dettenkofer M, Panning M. Characterisation of nosocomial and community-acquired influenza in a large university hospital during two consecutive influenza seasons. J Clin Virol 2015;73:47-51.

31. Jaeger JL, Patel M, Dharan N, et al. Transmission of 2009 pandemic influenza A (H1N1) virus among healthcare personnel-Southern California, 2009. Infect Control Hosp Epidemiol 2011;32:1149-1157.

32. Jhung MA, D’Mello T, Pérez A, et al. Hospital-onset influenza hospitalizations--United States, 2010-2011. Am J Infect Control 2014;42:7-11.

33. Jonges M, Rahamat-Langendoen J, Meijer A, Niesters HG, Koopmans M. Sequence-based identification and characterization of nosocomial influenza A(H1N1)pdm09 virus infections. J Hosp Infect 2012;82:187-193.

34. Khandaker G, Rashid H, Zurynski Y, et al. Nosocomial vs community-acquired pandemic influenza A (H1N1) 2009: a nested case-control study. J Hosp Infect 2012;82:94-100.

35. Lalayanni C, Sirigou A, Iskas M, Smias C, Sakellari I, Anagnostopoulos A. Outbreak of novel influenza A (H1N1) in an adult haematology department and haematopoietic cell transplantation unit: clinical presentation and outcome. J Infect 2010;61:270-272.

36. Launay E, Ovetchkine P, Saint-Jean M, et al. Novel influenza A (H1N1): clinical features of pediatric hospitalizations in two successive waves. Int J Infect Dis 2011;15:e122-e130.

37. Leckerman KH, Sherman E, Knorr J, Zaoutis TE, Coffin SE. Risk factors for healthcare-associated, laboratory-confirmed influenza in hospitalized pediatric patients: a case-control study. Infect Control Hosp Epidemiol 2010;31:421-424.

38. Levin-Rector A, Nivin B, Yeung A, Fine AD, Greene SK. Building-level analyses to prospectively detect influenza outbreaks in long-term care facilities: New York City, 2013-2014. Am J Infect Control 2015;43:839-843.

39. Liu C, Schwartz BS, Vallabhaneni S, et al. Pandemic (H1N1) 2009 infection in patients with hematologic malignancy. Emerg Infect Dis 2010;16:1910-1917.

40. Liu YX, Jia N, Suo JJ, et al. Assessment of the risk of nosocomial 2009 H1N1 influenza infection among obstetric care workers. Int J Gynaecol Obstet 2011;112:140-141.

41. Lobo RD, Oliveira MS, Garcia CP, Caiaffa Filho HH, Levin AS. Pandemic 2009 H1N1 influenza among health care workers. Am J Infect Control 2013;41:645-647.

42. Loeb M, Dafoe N, Mahony J, et al. Surgical mask vs N95 respirator for preventing influenza among health care workers: a randomized trial. JAMA 2009;302:1865-1871.

43. Macesic N, Kotsimbos TC, Kelly P, Cheng AC. Hospital-acquired influenza in an Australian sentinel surveillance system. Med J Aust 2013;198:370-372.

44. Mahmud SM, Thompson LH, Nowicki DL, Plourde PJ. Outbreaks of influenza-like illness in long-term care facilities in Winnipeg, Canada. Influenza Other Respir Viruses 2013;7:1055-1061.

45. Marzano A, Marengo A, Ruggiero T, et al. Clinical impact of A/H1/N1/09 influenza in patients with cirrhosis: experience from a nosocomial cluster of infection. J Med Virol 2013;85:1-7.

46. Miller DR, Christie GL, Molyneaux P, Currie GP. An outbreak of H1N1 influenza in a respiratory unit. Thorax 2010;65:938-939.

47. Milupi M, Madeo M, Brooke N, Ahmad SJ. Neonatal influenza A/H1N1/2009 outbreak in a UK district general hospital. J Hosp Infect 2012;81:131-133.

48. Mitchell R, Taylor G, McGeer A, et al. Understanding the burden of influenza infection among adults in Canadian hospitals: a comparison of the 2009-2010 pandemic season with the prepandemic and postpandemic seasons. Am J Infect Control 2013;41:1032-1037.

49. Moore C, Galiano M, Lackenby A, et al. Evidence of person-to-person transmission of oseltamivir-resistant pandemic influenza A(H1N1) 2009 virus in a hematology unit. J Infect Dis 2011;203:18-24.

50. Nivin B, Stoute A. Detection of nosocomial influenza outbreaks in long-term care facilities through active laboratory surveillance in New York City. Am J Infect Control 2008;36:498-499.

51. Oguma T, Saito R, Masaki H, et al. Molecular characteristics of outbreaks of nosocomial infection with influenza A/H3N2 virus variants. Infect Control Hosp Epidemiol 2011;32:267-275.

52. Pagani L, Thomas Y, Huttner B, et al. Transmission and effect of multiple clusters of seasonal influenza in a Swiss geriatric hospital. J Am Geriatr Soc 2015;63:739-744.

53. Pérez-Padilla R, Fernández R, García-Sancho C, et al. Demand for care and nosocomial infection rate during the first influenza AH1N1 2009 virus outbreak at a referral hospital in Mexico City. Salud Pública Méx 2011;53:334-340.

54. Pollara CP, Piccinelli G, Rossi G, et al. Nosocomial outbreak of the pandemic Influenza A (H1N1) 2009 in critical hematologic patients during seasonal influenza 2010-2011: detection of oseltamivir resistant variant viruses. BMC Infect Dis 2013;13:127.

55. Rabagliati R, Labarca J, Siri L, Perez CM, Ferrés M. Rates of hospital-acquired influenza due to the pandemic H1N1 virus in 2009, compared with seasonal influenza. Infect Control Hosp Epidemiol 2011;32:198-200.

56. Régis C, Voirin N, Escuret V, et al. Five years of hospital based surveillance of influenza-like illness and influenza in a short-stay geriatric unit. BMC Res Notes 2014;7:99.

57. Reid G, Huprikar S, Patel G, et al. A multicenter evaluation of pandemic influenza A/H1N1 in hematopoietic stem cell transplant recipients. Transpl Infect Dis 2013;15:487-492.

58. Riphagen-Dalhuisen J, Burgerhof JG, Frijstein G, et al. Hospital-based cluster randomised controlled trial to assess effects of a multi-faceted programme on influenza vaccine coverage among hospital healthcare workers and nosocomial influenza in the Netherlands, 2009 to 2011. Euro Surveill 2013;18:20512.

59. Risa KJ, McAndrew JM, Muder RR. Influenza outbreak management on a locked behavioral health unit. Am J Infect Control 2009;37:76-78.

60. Rodríguez-Sánchez B, Alonso M, Catalán P, et al. Genotyping of a nosocomial outbreak of pandemic influenza A/H1N1 2009. J Clin Virol 2011;52:129-132.

61. Rubin MS, Nivin B, Ackelsberg J. Effect of timing of amantadine chemoprophylaxis on severity of outbreaks of influenza a in adult long-term care facilities. Clin Infect Dis 2008;47:47-52.

62. Seale H, Weston KM, Dwyer DE, et al. The use of oseltamivir during an influenza B outbreak in a chronic care hospital. Influenza Other Respir Viruses 2009;3:15-20.

63. Spaeder MC, Fackler JC. Hospital-acquired viral infection increases mortality in children with severe viral respiratory infection. Pediatr Crit Care Med 2011;12:e317-e321.

64. Taylor G, Mitchell R, McGeer A, et al. Healthcare-associated influenza in Canadian hospitals from 2006 to 2012. Infect Control Hosp Epidemiol 2014;35:169-175.

65. Tsagris V, Nika A, Kyriakou D, et al. Influenza A/H1N1/2009 outbreak in a neonatal intensive care unit. J Hosp Infect 2012;81:36-40.

66. Valley-Omar Z, Nindo F, Mudau M, Hsiao M, Martin DP. Phylogenetic Exploration of Nosocomial Transmission Chains of 2009 Influenza A/H1N1 among Children Admitted at Red Cross War Memorial Children’s Hospital, Cape Town, South Africa in 2011. PloS One 2015;10:e0141744.

67. Vanhems P, Voirin N, Roche S, et al. Risk of influenza-like illness in an acute health care setting during community influenza epidemics in 2004-2005, 2005-2006, and 2006-2007: a prospective study. Arch Intern Med 2011;171:151-157.

68. Vayalumkal JV, Gravel D, Moore D, Matlow A, Canadian Nosocomial Infection Surveillance Program. Surveillance for healthcare-acquired febrile respiratory infection in pediatric hospitals participating in the Canadian Nosocomial Infection Surveillance Program. Infect Control Hosp Epidemiol 2009;30:652-658.

69. Veenith T, Sanfilippo F, Ercole A, et al. Nosocomial H1N1 infection during 2010-2011 pandemic: a retrospective cohort study from a tertiary referral hospital. J Hosp Infect 2012;81:202-205.

70. Vij NK, Stryker CC, Esper FP, Jacobs MR, Gonzalez BE. Influenza A/H1N1/09-10 infections in a NICU during the 2009-2010 H1N1 pandemic. Pediatrics 2011;128:e1297-e1301.

71. Voirin N, Payet C, Barrat A, et al. Combining high-resolution contact data with virological data to investigate influenza transmission in a tertiary care hospital. Infect Control Hosp Epidemiol 2015;36:254-260.

72. Weedon KM, Rupp AH, Heffron AC, et al. The impact of infection control upon hospital-acquired influenza and respiratory syncytial virus. Scand J Infect Dis 2013;45:297-303.

73. Wilkinson K, Mitchell R, Taylor G, et al. Laboratory-confirmed pandemic h1n1 influenza in hospitalized adults: findings from the Canadian Nosocomial Infections Surveillance Program, 2009-2010. Infect Control Hosp Epidemiol 2012;33:1043-1046.

74. Wise ME, De Perio M, Halpin J, et al. Transmission of pandemic (H1N1) 2009 influenza to healthcare personnel in the United States. Clin Infect Dis 2011;52 Suppl 1:S198-S204.

75. Wong BC, Lee N, Li Y, et al. Possible role of aerosol transmission in a hospital outbreak of influenza. Clin Infect Dis 2010;51:1176-1183.
