# Supplementary material for: Definition of healthcare‐associated influenza: A review and results from an international survey
Source: Influenza Other Respir Viruses. 2017 Jul 18;11(5):367–71. doi: 10.1111/irv.12460 (PMC5596525; doi:10.1111/irv.12460)
Supplement: Supplementary file 2 [file IRV-11-367-s002.docx]

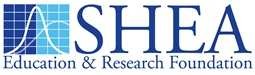


**NOSOFLU Project**

Healthcare-associated influenza (HA-flu) is associated with significant morbidity, mortality, and costs due to increased length of stay, but is likely to be under- recognized. Early detection in hospitalized patients leads to the initiation of respiratory isolation precautions and prevents nosocomial transmission to other patients as well as healthcare workers. Timely detection could also obviate extra testing and treatment.

The purpose of this survey is to learn about the definition of HA-flu used locally by hospitals of the SHEA Research Network (SRN) for the management of such infections. It may help in development of expert guidelines on HA-flu by contributing to a standardized definition.

**Email address:** (For de-duplication and removed by an administrator. This email address will not receive survey reminders. More than one may be entered.)

**SRN Institutional ID** (found on the email by "Participant Eligibility")**:**

**Hospital role/title:**

Infection preventionist


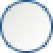


Hospital epidemiologist Nurse of infection control


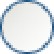

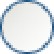


Other


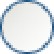


**Is your facility affiliated with an academic institution?**

Yes


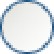


No


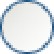


**Where applicable, please indicate the approximate number of beds in your hospital.**

Adult ICU Adult Surgical Adult Medical Obstetric Pediatric (all) Neonatal (all)


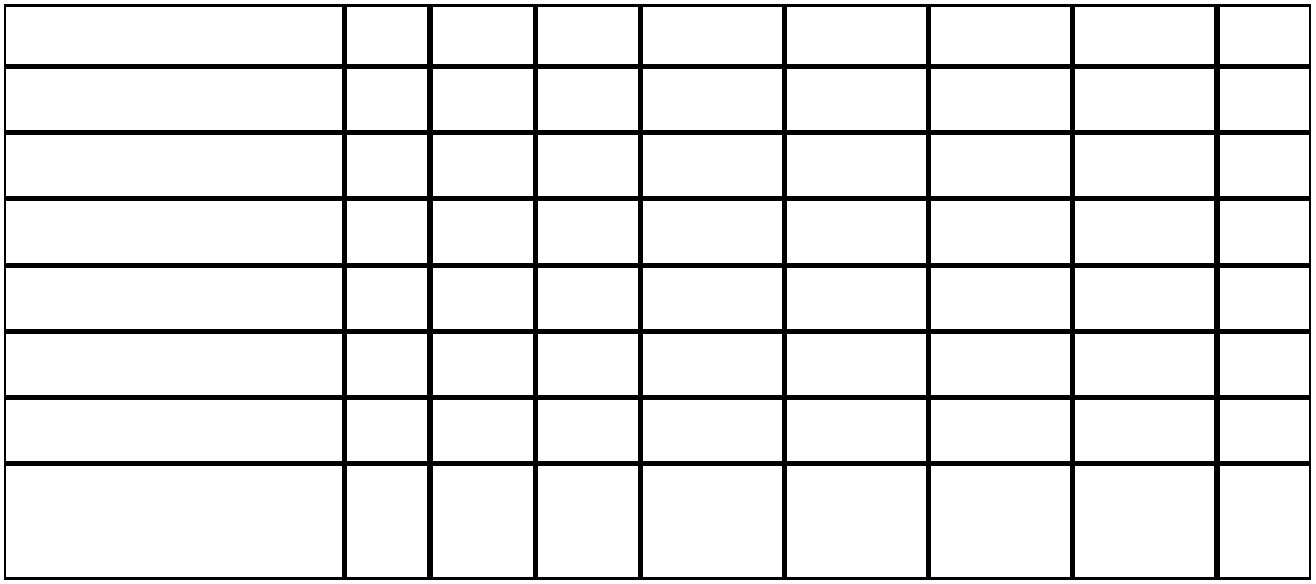

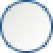

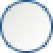

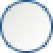

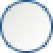

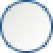

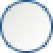

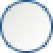

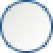

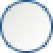

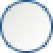

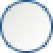

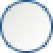

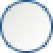

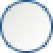

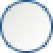

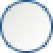

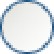

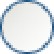

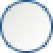

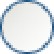

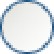

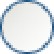

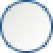

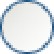

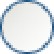

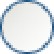

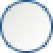

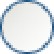

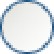

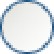

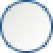

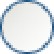

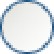

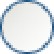

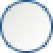

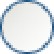

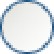

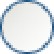

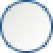

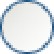

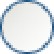

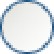

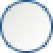

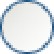

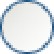

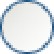

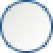

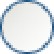

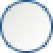

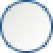

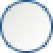

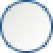

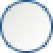

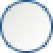

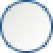

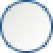


Other

5-24 25-29 30-99 100-199 200-299 300-399 400-499 >500

**A. Index Case** (first case or sporadic case)

**1. Do you use a standardized definition of healthcare-associated influenza (HA-flu)?**

Yes


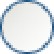


No


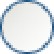


Don't know


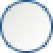


**2. If yes, your definition is based on:**

Clinical features Virologic features Both


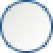

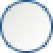

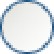


Don't know


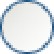


**3. Comments:**


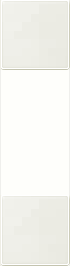


**Index Case: Definition of Clinical ILI Used at Your Institution**

The following questions are based on the following "natural history" of influenza like illness (ILI) and influenza.


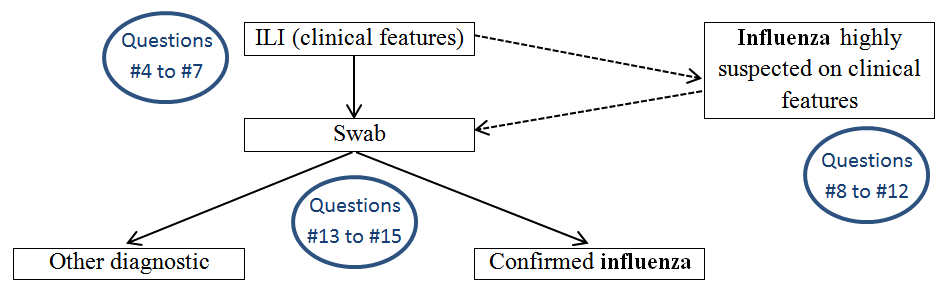


**4. Is fever part of the ILI definition?**

Yes


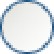


No


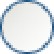


Don't know


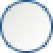


**5a. What is the threshold of fever for a PATIENT free of antipyretic? (°C)**

**5b. What is the threshold of fever for a HEALTHCARE WORKER free of antipyretic? (°C)**

**6a. What is the threshold of fever for a PATIENT with antipyretic? (°C)**

**6b. What is the threshold of fever for a HEALTHCARE WORKER with antipyretic? (°C)**

**7a. Which clinical features are present in the ILI PATIENT definition in your institution?** (click only those that apply)

*passage of 3 or more loose or liquid stools per day (WHO definition)

Required Symptom Optional Symptom

Chills

Cough

Sore throat

Nasal obstruction Rhinorrhoea (runny nose) Dyspnea (shortness of breath) Tachypenia (rapid breathing) Myalgia (muscle aches) Headache


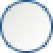

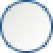

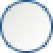

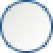

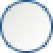

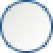

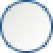

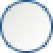

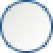

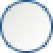

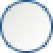

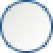

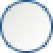

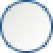

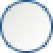

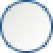

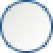

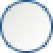

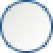

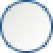

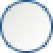

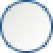

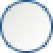

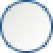


Asthenia (lack of energy/strength) Malaise

Poor feeding

Nausea and/or vomiting

Diarrhoea*

**7b. Which clinical features are present in the ILI HEALTHCARE WORKER definition in your institution?** (click only those that apply)

*passage of 3 or more loose or liquid stools per day (WHO definition)

Required Symptom Optional Symptom

Chills

Cough

Sore throat

Nasal obstruction Rhinorrhoea (runny nose) Dyspnea (shortness of breath) Tachypenia (rapid breathing) Myalgia (muscle aches) Headache

Asthenia (lack of energy/strength) Malaise

Poor feeding

Nausea and/or vomiting

Diarrhoea*

**If your institution's definition of diarrhoea differs from the WHO definition, please provide it here:**

**Please list any additional required or optional symptoms not listed above and indicate whether they apply to patients or healthcare workers.**

**Index Case: Definition of clinical INFLUENZA used in your institution before any virologic confirmation.**

**8. Do you use a particular definition to identify influenza with clinical features?**

Yes

No

Don't know

*If you answered no to the previous question, please go to question 13.*

**9. Is fever a part of the influenza definition?**

Yes

No

Don't know

**10a. What is the threshold of fever for a PATIENT free of antipyretic? (°C)**

**10b. What is the threshold of fever for a HEALTHCARE WORKER free of antipyretic? (°C)**

**11a. What is the threshold of fever for a PATIENT with antipyretic? (°C)**

**11b. What is the threshold of fever for a HEALTHCARE WORKER with antipyretic? (°C)**

**12a. Which clinical features are present in the influenza PATIENT definition in your institution?** (click only those that apply)

*in case of suspicion of hospital-acquired ILI** passage of 3 or more loose or liquid stools per day (WHO definition)

Required Symptom Optional Symptom

Chills

Cough

Sore throat

Nasal obstruction Rhinorrhoea (runny nose) Dyspnea (shortness of breath) Tachypenia (rapid breathing) Myalgia (muscle aches) Headache

Asthenia (lack of energy/strength) Malaise

Poor feeding

Nausea and/or vomiting

Diarrhoea**

**12b. Which clinical features are present in the influenza HEALTHCARE WORKER definition in your institution?** (click only those that apply)

*in case of suspicion of hospital-acquired influenza** passage of 3 or more loose or liquid stools per day (WHO definition)

Required Symptom Optional Symptom

Chills

Cough

Sore throat

Nasal obstruction Rhinorrhoea (runny nose) Dyspnea (shortness of breath) Tachypenia (rapid breathing) Myalgia (muscle aches) Headache

Asthenia (lack of energy/strength) Malaise

Poor feeding

Nausea and/or vomiting

Diarrhoea**

**If your institution's definition of diarrhoea differs from the WHO definition, please provide it here:**

**Please list any additional required or optional symptoms not listed above and indicate whether they apply to patients or healthcare workers.**

**Index Case: Diagnostic Tests Recommended in Your Institution**

**13. What type of swab is used?**

Nasal swab Throat swab Both

Don't know

Other

**14. What types of tests are performed?**

Viral isolation in cell culture/eggs

Molecular identification (reverse transcription-polymerase chain reaction (RT-PCR)) Antigen detection by immunofluorescence test (Rapid diagnostic test)

Serological test

Don't know

Other

**15. Are virologic samples sent to a national reference center for confirmation or other (strain, subtype, etc.)?**

Yes

No

Don't know

**Index Case: Definition of Healthcare-Associated INFLUENZA**

**16. What is the threshold delay between admission in the unit and the onset of symptoms used for definition of healthcare-associated influenza:** (in hours)

**17. Do you use other criteria to identify HA-flu?**

Yes

No

Don't know

**If yes, please explain:**

**18. Do you use adapted definition of healthcare-associated ILI and/or healthcare-associated influenza for specific patients?**

Yes

No

Don't know

**19. If yes, for patients with:**

Cancer

HIV/AIDS

Immunocompromised/ inflammatory diseases

Hematological diseases

Other

**20. Do you use different definition of healthcare-associated ILI and/or healthcare-associated influenza for healthcare workers compared with patients?**

Yes

No

Don't know

**If yes, please provide details:**

**B. Detection of Cluster**

**21. Do you use a standardized definition for a cluster of healthcare-associated influenza?**

Yes

No

Don't know

**22. Based on your local guidelines or personal experience how many cases define a cluster?**

**23. Are healthcare workers counted in the cluster?**

Yes

No

Don't know

**If no, please explain why:**

**24. Please explain on what case definitions your institution decides to begin a cluster investigation:**

Clinical features only

Don't know

Other (i.e. clinical features and virologic confirmation)

**25. Which geographical unit do you use for cluster investigation/definition?**

Same room Same unit Same floor

Same building

Other

**26. Which time interval do you consider to suspect cross-transmission between two cases?** (in hours)

**27. To confirm the cluster, do you search for a similarity of strains?**

Yes

No

Don't know

**If yes, what technique is used?**

**C. Notification**

**28. Do you report nosocomial INFLUENZA cases to other organizations?**

Yes

No

Don't know

**29. If yes, what are the criteria of notification?**

Number of cases (please provide below)

Number of deceased cases (please provide below)

Unexpected cases (outside the seasonal circulation of influenza, i.e. travel from southern hemisphere in summer for the northern hemisphere) Don't know

Other

**If the criteria is "number of cases," please list it here:**

**If the criteria is "deceased number of cases," please list it here:**

**30. To whom do you report nosocomial influenza cases?**

Your institution

Local public health authority

National public health authority

Local virologic reference center

National virologic reference center

Don't know

Other

**31. What actions are decided for your institution based on notification?**

**32. Comments**
